# Tailoring a Text Messaging and Fotonovela Program to Increase Patient Engagement in Colorectal Cancer Screening in a Large Urban Community Clinic Population: Quality Improvement Project

**DOI:** 10.2196/43024

**Published:** 2023-08-10

**Authors:** Monica Guo, Rena Brar Prayaga, Carly E Levitz, Elena S Kuo, Esmeralda Ruiz, Evelyn Torres-Ozadali, Anne Escaron

**Affiliations:** 1 Institute for Health Equity AltaMed Los Angeles, CA United States; 2 mPulse Mobile Inc. Encino, CA United States; 3 Center for Community Health and Evaluation Kaiser Permanente Washington Health Research Institute Seattle, WA United States

**Keywords:** colorectal cancer screening, texting program, fotonovela, fecal immunochemical test, FIT, FIT kit, thematic analysis, mobile phone

## Abstract

**Background:**

Appropriate annual screenings for colorectal cancer (CRC) are an essential preventive measure for the second-leading cause of cancer-related death in the United States. Studies have shown that CRC screening rates are influenced by various social determinants of health (SDOH) factors, including race, ethnicity, and geography. According to 2018 national data, participation in screening is lowest among Hispanic or Latinx individuals (56.1%). At an urban Federally Qualified Health Center, a quality improvement project was conducted to evaluate a texting program with a motivational fotonovela—a short narrative comic. Fotonovelas have previously been used in programs to improve knowledge of cervical cancer and human papillomavirus, vaccinations, and treatments for depression.

**Objective:**

This study aimed to encourage compliance with fecal immunochemical test (FIT) screening. Patient engagement involved a texting program with fotonovelas informed by behavior change techniques. This study sought to understand the qualitative characteristics of patient motivation, intention, and barriers to completing their screening.

**Methods:**

A total of 5241 English-speaking or Spanish-speaking Federally Qualified Health Center patients aged 50 to 75 years were randomized to either intervention (a 4-week tailored 2-way texting program with a fotonovela comic) or usual care (an SMS text message reminder and patient navigator phone call). The texting vendor used a proprietary algorithm to categorize patients in the intervention group into SDOH bands based on their home addresses (high impact=high social needs and low impact=low social needs). Over 4 weeks, patients were texted questions about receiving and returning their FIT, what barriers they may be experiencing, and their thoughts about the fotonovela.

**Results:**

The SDOH index analysis showed that most of the patient population was in the SDOH band categories of high impact (555/2597, 21.37%) and very high impact (1416/2597, 54.52%). Patients sent 1969 total responses to the texting system. Thematic analysis identified 3 major themes in these responses: messages as a reminder, where patients reported that they were motivated to return the FIT and had already done so or would do so as soon as possible; increasing patients’ understanding of screening importance, where patients expressed an increased knowledge about the purpose and importance of the FIT; and expressing barriers, where patients shared reasons for not completing the FIT.

**Conclusions:**

The texting program and fotonovela engaged a subset of patients in each SDOH band, including the high and very high impact bands. Creating culturally tailored messages can encourage patient engagement for accepting the content of the messaging, confirming intentions to complete their FIT, and sharing insights about barriers to behavior change. To better support all patients across the continuum of care with CRC screening, it is important to continue to develop and assess strategies that engage patients who did not return their home-mailed FIT.

## Introduction

### Background

Colorectal cancer (CRC) is the second-leading cause of cancer-related deaths in the United States, accounting for an estimated 53,200 deaths in 2020 [1]. CRC is mostly preventable with appropriate screening and can be treated successfully (5-year survival rate of approximately 90%) when found at early stages [1]. Fecal immunochemical test (FIT) is a screening tool for CRC, which has shown promise in increasing screening and early detection rates [2].

### Social Determinants of Health and CRC Screening

Studies have shown that CRC screening rates are influenced by various sociodemographic factors, such as race and ethnicity, socioeconomic status (SES), and geography [3]. According to 2018 national data, participation in screening is lowest among Hispanic or Latinx individuals (56.1%), followed in order by American Indian or Alaska Native (62.1%), Asian and Pacific Islander (64.8%), Black (70%), and White (71%) individuals [3]. Among Hispanic or Latinx individuals, factors that affect screening rates include SES, language barriers, health literacy, education, undocumented status, lack of insurance, and limited access to health care services [3]. According to the Wisconsin County Health Rankings surveys, socioeconomic factors of education, income, and social disruption are 40% of the factors that influence health determinants [4], which underscores their contribution to health outcomes.

Specifically regarding SES, higher screening rates are seen in those with higher income [5]. In California, the 2018 Behavioral Risk Factor Surveillance System reported that 74% of insured residents completed CRC screening compared with 45% of uninsured residents [6]. Although factors such as SES can be confounded by race and ethnicity, studies show that these remain as factors even when controlling for SES effects in screening rates by race and ethnicity [3].

### Use of Fotonovela as a Health Literacy Tool

A visual narrative approach using fotonovelas—comics that impart a message—has been piloted with a wide range of users and shows promise for narrowing the health literacy gap for underserved or marginalized populations [7-9]. Fotonovelas have been used in programs to improve knowledge about cervical cancer and vaccinations [7,9] and treatments for depression [10]. A 2019 study of an intervention using a print fotonovela for increasing CRC screening found that the intervention group had a higher rate of FIT completion than the comparison group, although the difference was not statistically significant [11]. A study by Thompson et al [9] is one of the few that developed a cancer risk fotonovela in a digital medium and curated it for a Latina patient population. Other digital uses of fotonovelas have included interventions to encourage COVID-19 vaccination adoption [12].

### Texting in CRC Interventions

Reviews of mobile health interventions and their impact on cancer screening have found that these interventions increase screening rates, with larger increases in interventions that combine multiple modes of engaging patients [13,14]. This increase has been demonstrated in underserved patients and patients with English as a secondary language in the United States [15]. CRC interventions that use texting primarily use text messages as reminders for patients to complete their FIT and then combine the reminders with other modes of outreach, such as automated and live phone calls [14].

When SMS text message interventions result in a high volume of responses from patients, the use of machine learning and natural language understanding (NLU) can make it possible to respond to patients where the workload was too heavy before. They achieve this by categorizing and automating certain types of responses [16]. These tools provide quick, automated replies to a patient’s questions and responses, without the need to wait for a staff member’s involvement.

There is an opportunity to contribute to studies that look at screening uptake by social determinants of health (SDOH). According to the 2021 American Community Survey, 40% of low-income households do not have a mobile phone data plan, and among older adults, only approximately 50% own a smartphone [17]. This is relevant to patients eligible for CRC screening, which skews toward the age range of 50 to 75 years. In this Federally Qualified Health Center (FQHC) population, we previously reported that those who engaged in a texting program were more likely to have increased social needs [18]. In this paper, we further explored the behaviors that could explain this relationship through qualitative data analysis of patients’ SMS text message responses.

Patients face barriers to completing their FIT kits, which likely differ according to cultural context and other factors. Reviews have noted that there is a gap in the literature regarding barriers to using mobile health among older adult patients [19], although other studies have found that older adults are open to these text messages [20]. Previous qualitative analysis conducted internally at the FQHC found that 66% of interviewed patients (n=27) said that the screening was “scary because it elicited thoughts about life and death” [21]. These findings directly informed the messaging and educational information of this quality improvement project.

### Objectives

This study describes the development of a culturally tailored digital fotonovela and bidirectional SMS text messaging program for CRC screening, in which we measured patients’ level of engagement and reviewed patient qualitative feedback on fotonovela acceptability. Additional results are intended to provide insights into the relationship between SDOH and the level of patient engagement.

This paper also reports on the design and development of bidirectional texting and digital fotonovelas as a collaborative, iterative process between the FQHC and the text message platform vendor mPulse Mobile.

## Methods

### Fotonovela Development

This quality improvement project was conducted in a large urban FQHC. Digital fotonovelas were developed by the texting vendor based on prior internal work at the FQHC [18]. The illustrations and storyline of the fotonovelas focused on depicting role models who were identified as peers of the population of interest: Hispanic or Latinx patients aged 50 to 75 years from a large urban FQHC, with similar cultural and social norms to communicate the message in English or Spanish.

The FQHC’s CRC clinical team and the texting vendor collaborated on the narrative and visual content of the fotonovelas. The vendor brought expertise in behavioral science and experience creating similar programs. The FQHC team brought literature on screening barriers and knowledge about their specific patient population. A series of meetings led to 2 drafts: 1 for male participants and 1 for female participants. Revisions to the drafts included adding cultural elements to better reflect the appearances, practices, and preferences of FQHC age-eligible patients and to intensify user engagement and emotional connectedness with culturally aligned characters.

**Figure 1:**
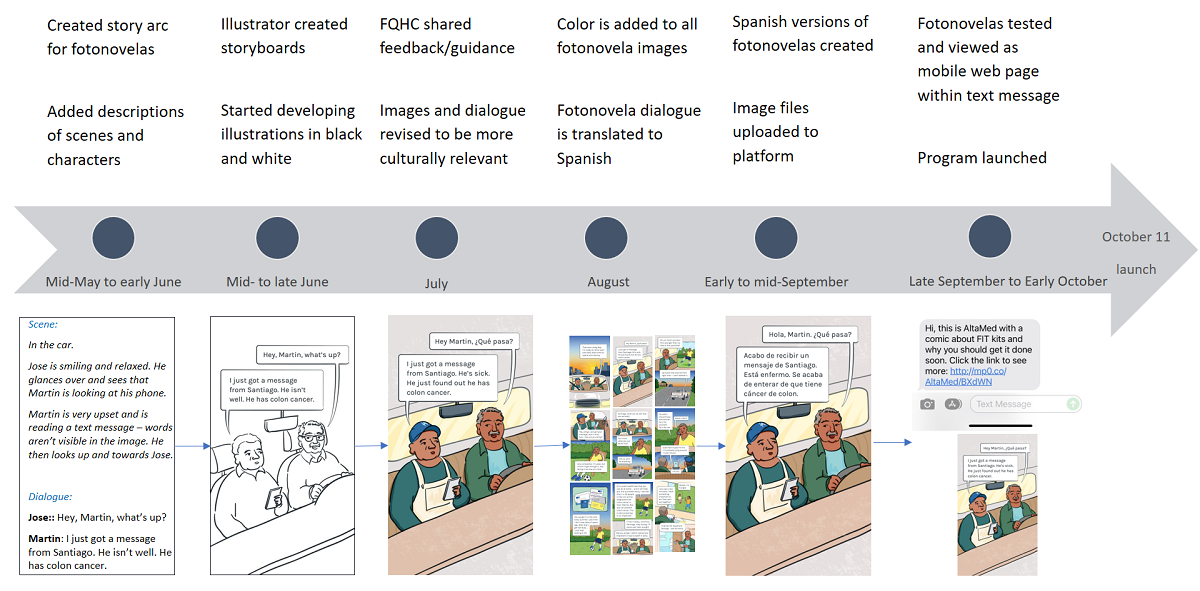
Fotonovela development process for the fotonovela tailored for the male patient population. FQHC: Federally Qualified Health Center.

For example, the evolution of the fotonovela titled *Turning 50* (tailored for women) included changing the main characters’ hairstyle and skin color and changing the beverage from tea to coffee. Other enhancements included adding images of family within the storyline, such as a child holding her mother’s hand, and a mix of English and Spanish languages reflecting an informal setting (refer to Figure 1 for second fotonovela).

**Figure 2:**
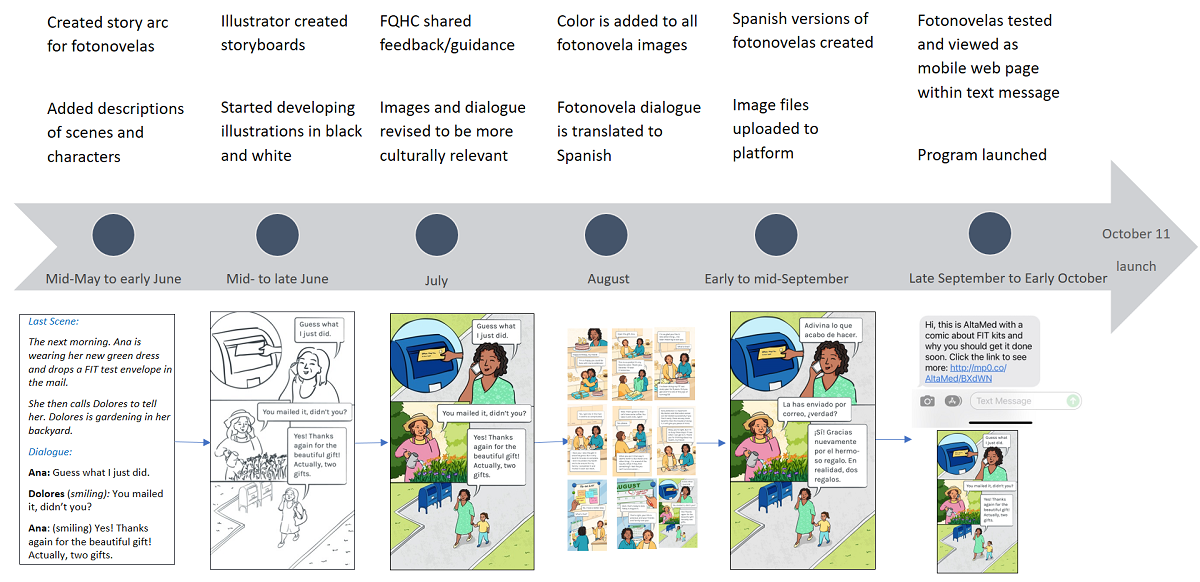
The 2021 timeline describing steps taken to develop the colorectal cancer screening fotonovela for female participants aged 50 to 75 years. FQHC: Federally Qualified Health Center.

The final fotonovelas were translated into Spanish and saved as images. These images were uploaded to the vendor’s platform, where both teams tested and revised the fotonovelas before launching the text message program. Figure 2 shows the 2021 timeline for both fotonovelas, with the version for female participants presented alongside the timeline.

The fotonovela link was texted as part of the 4-week texting program, which drew from the literature on increasing cancer screening rates. The first series of text messages confirmed that patients had received the FIT kit in the mail and then recommended setting a goal (“Complete the test” and “Mail it back in the next 2 days”). Subsequent messages were aimed at building health literacy (“It can help us find colon cancer early”) and eliminating doubts about whether it was necessary (“We know you’ve completed colon cancer screening before, but you are due now”). Some barriers were addressed explicitly by a text sent during the second week of messaging that asked patients, “If you haven’t done your kit yet, please tell us if any of these reasons apply (please pick your most important reason).” Patients chose from a list of (1) I’m not sure why I need it; (2) I feel fine, and I don’t have any pain or symptoms; (3) I’m too busy right now; (4) I’m scared about the results; (5) It’s embarrassing to do it and then mail it back; and a final option to text back with barriers in their own words. Other barriers were addressed, as presented in Multimedia Appendix 1, using NLU to reply to patient concerns with automated text messages.

Behavior change strategies and techniques, such as building self-efficacy, positive framing, goal setting, verbal persuasion, and addressing myths and misconceptions, were embedded within texts to patients. The text messages addressed the most common barriers to completing CRC screening, referenced the importance of family and consulting family in decision-making, explained not putting off the screening because it was uncomfortable or unfamiliar, and reinforced the importance of early cancer detection. The texting medium was thought to provide a level of openness and distance that would encourage patients to feel comfortable sharing information or asking questions that they might otherwise not pose in person, such as how to collect a sample from their stool. The teams designed the fotonovela to incorporate behavior change techniques consistent with patients’ receptiveness to screening and to anticipate and address potential patient barriers, as presented in Multimedia Appendix 1.

### NLU Classification

When patients texted back replies, the automated responses used NLU and conversational artificial intelligence (AI) to classify patient replies into expected categories and send appropriate follow-up messages. For example, to recognize that patients were avoiding the screening because they were worried about the results, the system looked for the following terms in text messages: “scared,” “worried,” “dread,” “anxious,” “panic,” “frightened,” “agitated,” or “afraid.” The system addressed these emotions in text replies such as, “We understand that it can be stressful to wait for test results. But the results are so important because they tell you about your colon health. The good thing about catching problems early is that they can be treated.” There were 35 conversational AI- or NLU-based rules in place for many other anticipated themes in patient messages, such as procrastination, disgust, completed colonoscopy in the past, confusion about how to complete the screening, and not being clear about why it was necessary.

### Bidirectional Texting Program Implementation—Mailing Kits

Since 2016, the FQHC has conducted an annual FIT mailing for patients who were overdue for CRC screening. Patients received an FIT kit in the mail at their home address on file, with written instructions (in English or Spanish based on the preferred language) and visual instructions on how to complete their FIT and return it in the mail. Building on the learnings from the Participatory Research to Advance Colon Cancer Prevention study [21], the 2021 FIT kit mailing targeted patients with (1) at least 1 clinic visit in the last 2 years (since July 2019); (2) a phone number on file; (3) an FIT kit completion status of “Never completed,” “Last completed within 12-24 months,” or “Last completed more than 24 months ago”; and (4) no gastrointestinal referral associated with rectal bleed symptoms.

In July 2021, a total of 11,000 eligible patients were mailed the FITs. All patients received the usual care clinical workflow to encourage FIT completion. First, patients received 2 automated texts from the FQHC’s internal system: one primer before the mailing and another reminding them to return their FIT. In August, patient navigators called patients who had yet to return an FIT, answered any questions about the FIT, and encouraged screening test completion. At the end of September, 5241 patients had still not completed their FIT and were randomized for the next step of the quality improvement project that was previously described. Patients were block randomized by binary sex (male or female), age group (50-60 years and 61-75 years), and prior screening history [18].

The extent to which patients interacted with the program was characterized by 2 measures: engagement rate and time to respond to week 1 of the texting program. The engagement rate was calculated by taking the total number of unique patients who interacted with the system (text responses or clicks to links sent) without opting out and dividing it by the total number of unique intervention group patients outreached. The time taken by the patient to respond in week 1 of the program was calculated as the percentage of responses within the first minute that the texts were sent, within 10 minutes, and within the first hour.

### Qualitative Data Analysis of Patients’ Free-Text Responses Throughout Intervention

Data analysis was conducted only for patients in the intervention group. Over the course of 4 weeks, the patients received 2 to 4 texts weekly from the system. The program included questions, reminders, and opportunities for patients to engage in bidirectional texting with the system, that is, respond to the texts received. The patients were also able to text back unprompted at any time. Weekly thematic analysis was conducted on all the received texts to provide timely feedback to the FQHC team to act on.

At the end of the quality improvement project, these free-text responses were all translated into English, if needed, and then analyzed as a whole to identify broad themes, subthemes, and anonymized illustrative quotes. These responses informed the project on whether the patients found the program and fotonovelas acceptable, engaging, and culturally relevant. Each patient’s quote was categorized by the SDOH band to provide more context to the patient’s response. The number of responses that expressed a particular theme and subtheme were tallied, allowing a single patient to provide multiple responses, both within 1 theme and across multiple themes. Subthemes with a small N value were still included because of the value of the information being shared and the FQHC’s interest in using the feedback to inform program changes.

### Quantitative Data Analysis—SDOH

In the intervention group, patient home addresses were run through the texting vendor’s proprietary algorithm to assign an SDOH index score (0-100) for each patient, where 0 represents a low needs census tract and 100 represents a high needs tract [22]. On the basis of the index score, the patient scores were sorted into 5 SDOH bands: very low impact (0-20), low impact (20-40), medium impact (40-60), high impact (60-80), and very high impact (80-100). If addresses were not recognized by the system, then the patient was placed in the unknown SDOH impact category. The patients’ home addresses were plotted on a map to visually describe the SDOH gradient and language preference (English or Spanish) among those assigned to a clinic in Los Angeles or Orange County. The response to SMS text messaging engagement by the SDOH impact band and by patient-preferred language was previously reported [18].

### Ethical Considerations

The Kaiser Permanente Washington Human Subjects Review Office reviewed and determined that this quality improvement project did not involve research and was therefore exempted from full review.

### Telephone Consumer Protection Act Health Care Exemption

The texting program also adhered to the Telephone Consumer Protection Act health care exemption, under which health plan members and patients who provide a mobile number implicitly consent to receive phone calls or text messages related to their health. All communication must offer an easy opt-out (text STOP), and opt-out requests must be honored immediately.

## Results

### SDOH Distribution

**Figure 3:**
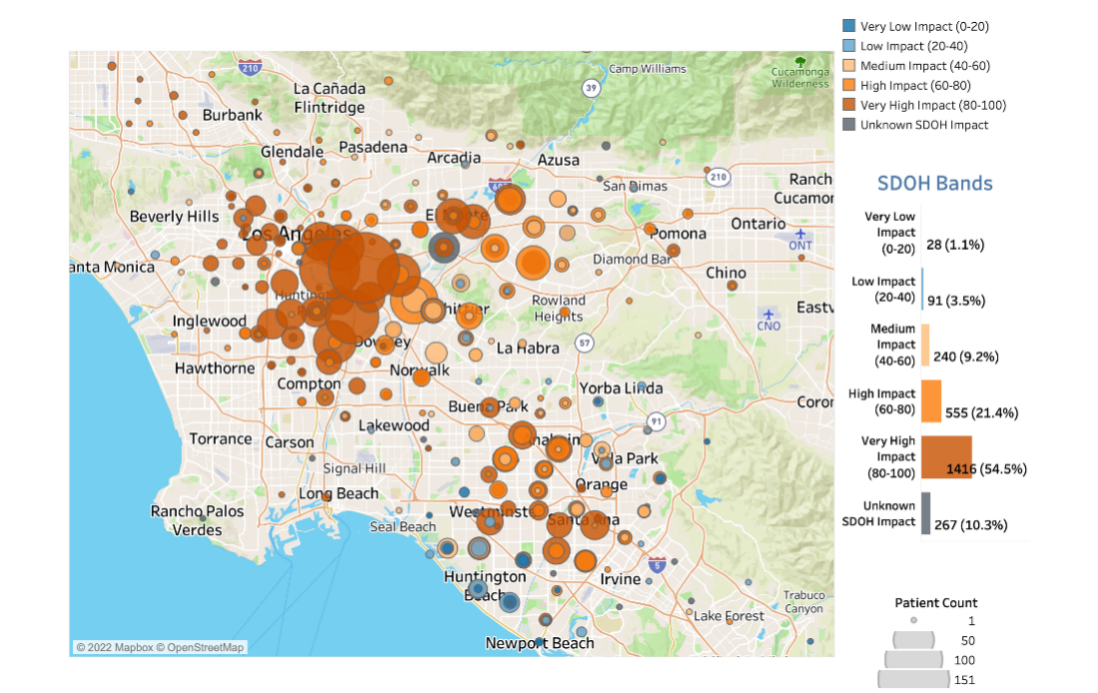
Map of intervention group patients eligible for colorectal cancer screening by social determinants of health (SDOH). The circle size represents number of people by zip code and color indicates level of SDOH impact (SDOH Index is 0 to 100). If a circle has more than one color, there is varying SDOH impact (census tract level) within the same zip code.

**Figure 4:**
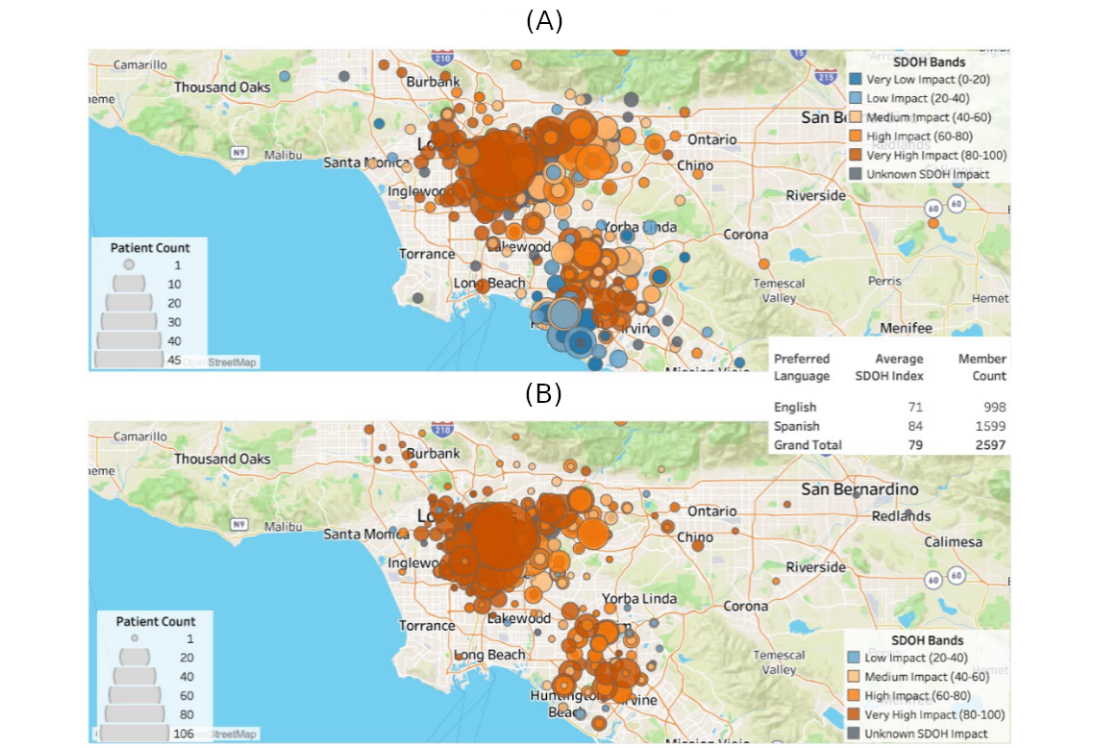
Map of intervention group patients eligible for colorectal cancer screening by social determinants of health (SDOH) and language preference. (A) SDOH Index by Language: English. (B) SDOH Index by Language: Spanish.

For this project, 2644 patients were randomized to the usual care group, whereas 2597 patients were randomized to the intervention group to receive the usual care plus text messages and fotonovela. Of the 2597 intervention patients with an address, 2330 were sorted into an SDOH band. Most of the patient population were in the high and very high impact bands (555/2597, 21.37% and 1416/2597, 54.52%, respectively; Figure 3). The average SDOH index score was higher among patients whose preferred language was Spanish (84) compared with English (71; Figure 4).

The overall engagement rate with the texting program was 39.51% (1026 patients responded to at least 1 text out of the 2597 patients in the intervention group) [18]. An analysis of responses for week 1 revealed that patients who responded (n=509) typically did so very quickly: 27.3% (139/509) within the first minute, 53.4% (272/509) within 10 minutes, and 86.1% (438/509) within the first hour.

A total of 1969 responses were received from the patients and analyzed. Three major themes emerged:

1. Messages as a reminder: patients were motivated to return the FIT and had already done so or would do so as soon as possible.
2. Increasing patients’ understanding of screening importance: the intervention increased patients’ knowledge about the purpose and importance of the FIT, and patients changed their minds to express readiness to complete the FIT.
3. Expressing barriers: patients shared barriers to or reasons for not completing the FIT.

### Theme 1—Messages as a Reminder

This theme reflects that patients were motivated to return their FIT and had already done so or would do so as soon as possible. Some patients had positive intent and behavior toward completing their FIT and a renewed commitment to complete it, partly because of the reminders. Although most patients planned to mail back their tests, a proportion of patients expressed a preference to return their tests in person at the clinic. Subthemes from patients’ responses were returned kit already, plan to complete soon, will pick up at clinic, and will drop off at doctor’s office.

Table 1 summarizes the subthemes and shows example quotes from patients. For more example quotes, a full view of the table is available in the Multimedia Appendix 2.

**Table 1:** Theme 1: messages as a reminder.

Subtheme			Example quote
**Returned kit already (n=209)**			
	These patients returned their kits after receiving the reminder or, in some cases, even before the reminder messages.	“I totally forget it when I go to use the toilet [1 week later] I sent mine in already.” [Male, English speaker, aged 57 years, very high SDOH^a^ impact] “Yes, I already took it to the clinic.” [Female, Spanish speaker, aged 62 years, very high SDOH impact]	
**Plan to complete soon (n=88)**			
	These patients viewed the reminder as a call to action. They were not averse to completing the screening but needed a nudge to move forward. Sentiment was positive, and a few patients apologized for the delay in getting this done.	“Yes, we totally forgot. Will do. Thanks!” [Female, English speaker, age 73 years, low SDOH impact] “No I haven‘t yet I have one though, I‘ll get it done asap.” [Male, English speaker, aged 57 years, very high SDOH impact] “Yes, thanks, I’ll return it, don’t worry.” [Female, Spanish speaker, aged 68 years, very high SDOH impact]	
**Will pick up at clinic (n=25)**			
	Others (who did not already have a kit) were keen to move things along by picking up an FIT^b^ kit at a nearby clinic instead of waiting to receive one in the mail.	“No...I can drive there & pick one up.” [Male, English speaker, aged 62 years, medium SDOH impact] “I’m going in to [FQHC^c^] today, I’ll pick one up.” [Female, English speaker, aged 64 years, high SDOH impact] “I have not received one in the mail. I actually have an appointment this afternoon. Can I pick one up?” [Female, English speaker, aged 51 years, Unknown SDOH impact]	
**Will drop off at doctor’s office** **(n=8)**			
	These patients might be more comfortable with delivering the kit to their doctor. The provider might have encouraged or recommended the screening and would be able to answer any questions relating to results of the screening).	“I have an appt with Dr. [Redacted] on the 10/25, and will drop off the test at that time.” [Female, English speaker, aged 68 years, medium SDOH impact] “I was with my doctor and there they gave me the paper for the test so that I could take it to my next appointment which is this month.” [Female, Spanish speaker, aged 70 years, very high SDOH impact] “You mean the brush stool kit? I will bring with me on my doctor’s visit tomorrow.” [Male, English speaker, aged 55 years, high SDOH impact]	

^a^SDOH: social determinants of health.

^b^FIT: fecal immunochemical test.

^c^FQHC: Federally Qualified Health Center.

### Theme 2—Increasing Patients’ Understanding of the Importance of Screening

Patients expressed that the program content was helpful in understanding the preventive value of screening. Furthermore, subthemes (Table 2) showed that messaging and fotonovela content played a part in helping patients change their minds, complete their FIT, or rekindle interest in completing it.

**Table 2:** Theme 2: increasing the patients’ understanding of screening importance.

Subtheme			Example quote
**Changed mind because of text message or fotonovela content (n=46)**			
	An important goal of the program was to change behavior by explaining why the screening was important—both in the text messages and in the fotonovela scenes and dialog.	“I feel good I don’t feel symptoms, I feel good [1 week later] Thanks for convincing me. It convinces me. My mother suffers from the colon. I’ll take it Thursday.” [Male, Spanish speaker, aged 62 years, very high SDOH^a^ impact] After viewing fotonovela and being asked “Are you more likely to get [your FIT^b^] done this week after reading the comic?” “Yes I want to do it thanks.” [Female, Spanish speaker, aged 57 years, high SDOH impact].	
**Misplaced or ignored the kit but interest is now rekindled (n=26)**			
	These patients lost or threw away the kit and forgot all about it but are now interested in getting a new FIT kit and completing the screening.	“I’m not sure I will have to look for the kit. Is it still good or do I need a new one?” [Female, English speaker, aged 57 years, very high SDOH impact] “I don’t have it anymore... [2 weeks later] Please send me a new kit and I will complete it thank you.” [Female, English speaker, aged 58 years, very low SDOH impact] “It got thrown in trash by accident. Please send another.” [Male, English speaker, aged 65 years, high SDOH impact]	
**Understands preventive value of screening (n=12)**			
	After viewing the fotonovela, patients were asked why they would be more likely to complete the test and return the kit. The broad theme was to protect health and prevent cancer.	“Yes thank you for caring about me and I returned that.” [Female, Spanish speaker, aged 68 years, very high SDOH impact] “To prevent cancer.” [Male, Spanish speaker, aged 58 years, very high SDOH impact] “It’s better to prevent.” [Male, Spanish speaker, aged 59 years, high SDOH impact] “For my own health and safety.” [Male, English speaker, aged 57 years, very high SDOH impact]	
**Willing to redo test if lost in the mail (n=3)**			
	The importance of the screening was clear to patients like this who requested another kit and were willing to redo the test as it might have been lost in the mail.	“I got the kit, filled the kit and mailed some time ago. The mail is horrible here. We have constant problems. Send me another kit and will try again.” [Female, English speaker, aged 71 years, high SDOH impact]	

^a^SDOH: social determinants of health.

^b^FIT: fecal immunochemical test.

### Theme 3—Expressing Barriers

This last theme reflected that patients were willing to share barriers and explanations for why they had not completed the screening. Subthemes in Table 3 included patients who replied that they did not receive an FIT and who were then mailed another one by the care team. Other subthemes were patients who have health or mobility issues, who faced barriers and tradeoffs in terms of the physical requirements for being able to complete the kit; patients who were planning on getting a different screening; patients who were putting off or avoiding because it is unpleasant; and patients who shared reasons why they had other priorities competing for their attention.

There was also a subset (n=10) of responses where patients stated that they were not interested in getting screened. For the full list of example quotes from patients, see table in the Multimedia Appendix 3.

**Table 3:** Theme 3: expressing barriers.

Subtheme		Example quote
**Did not receive an FIT^a^ kit (n=290)**		
	These patients did not receive the FIT kit in the mail and were requesting another kit to complete and send back. The tone was generally polite and positive.	“I don’t have a package 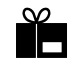 for the test.” [Male, Spanish speaker, aged 60 years, very high SDOH^b^ impact] “I have not received the FIT kit. Please mail it to me and I’ll complete it.” [Female, English speaker, aged 63 years, unknown SDOH impact]
**Have health or mobility issues** **(n=16)**		
	These patients had health issues and needed assistance, more time, or a good reason to complete the test.	“Well it might be quick and easy for you that have 2 working arms and legs. It’s difficult for me to balance.” [Female, English speaker, aged 56 years, very low SDOH impact] “I had a car accident and I had surgery on my leg and it is very painful.” [Female, Spanish speaker, aged 51 years, very high SDOH impact]
**Planning to get a different colon screening (n=13)**		
	The FIT test was not appropriate in these cases because they had recently completed a colonoscopy (or had one scheduled soon).	“Had a colonoscopy last month. They said I didn’t need to do that until next year!” [Female, English speaker, aged 59 years, very high SDOH impact]
**Not interested in the screening** **(n=10)**		
	These patients were not open to influence or persuasion and made it clear that they would not do the test.	“I don’t want you to send me one.” [Female, Spanish speaking, aged 57 years, very high SDOH impact] “I don’t want to do it.” [Female, Spanish speaking, aged 58 years, very high SDOH impact]
**Putting off or avoiding because it is unpleasant** (**n=9)**		
	These patients found the test disgusting or unpleasant but might also be confusing the FIT test with preparation for a colonoscopy.	“I can’t stomach drinking the solution that clears the intestines. It is a painful process that my body won’t allow me to go through with it.” [Male, English speaker, aged 60 years, very high SDOH impact] “Because it disgusts me to see that test, I’m going to do it.” [Female, Spanish speaker, aged 53 years, medium SDOH impact]
**Understands importance but life gets in the way (n=6)**		
	These patients took the time to explain why they were putting off completing the test and shared a mix of family concerns and other commitments.	“I am so busy packing I am moving to a smaller apt. Everything is everywhere. I will worry about this after the new year” [Female, English speaker, aged 62 years, low SDOH impact] “No, I have been taking care of my mom I’m sorry” [Male, English speaker, aged 61 years, very low SDOH impact] “I’m focused on a professional exam. Excuse me, tonight I complete it.” [Male, Spanish speaker, aged 67 years, high SDOH impact] “I haven’t had a chance to see it. I’ve got other big worries right now financially and I’m on a mission, I’ll get back with you shortly.” [Male, English speaker, aged 52 years, high SDOH impact]

^a^FIT: fecal immunochemical test.

^b^SDOH: social determinants of health.

## Discussion

### Principal Findings

In this quality improvement project, we described the steps to create a culturally tailored bidirectional text messaging program with fotonovelas for underserved patients to motivate the return of mailed CRC screening kits. The FQHC team and the texting vendor partnered on the iterative development of the texting program prompts and responses, including the creation of a digital fotonovela. We found that this type of culturally relevant messaging engaged English- and Spanish-speaking patients from every SDOH band. Patients responded to the messages, showing increased knowledge of the severity of CRC and their intentions to complete their FIT. They were also engaged in sharing personal health reasons and life situations for not returning their FIT.

We observed a 40% engagement rate in our primarily Medicaid population, which was higher than the engagement rates in vendors’ other health care texting programs of 10% to 20% [22,23]. Interestingly, there was no increase in attrition or patient opt-outs at the 4-week point, which suggests that the outreach struck an appropriate balance between too few and too many messages, and the perceived relevance and value of automated messaging remained high.

Messaging patients at multiple time points over the course of a few months was highly valuable. Patients could text back to say that they did not have an FIT, and the data were shared weekly with the FQHC staff, allowing them to mail new FIT kits to patients in a timely manner. Patients also texted back to share why they could not complete the FIT, which allowed for tailored motivational responses to be texted back. Most reasons were aligned with known barriers, and we plan to continue our usual care in response, including patients having existing plans to get a different colon screening and not receiving an FIT kit the first time. We used patient-reported screenings to retrieve medical records and update patient screening history for patients who reported having other screening plans, and we remailed patients who did not receive an FIT kit the first time.

One barrier that we did not know of was the health and mobility issues shared by patients. We have made our patient navigators aware of this barrier.

Our analysis of engagement by SDOH bands yielded several interesting results that could support future research. The maps illustrated that the intervention group patients spanned across the SDOH index and were drawn from both preferred language groups. Our project previously reported that the intervention resulted in patients who were engaged across all SDOH bands, from high to low social needs, with few patients opting out (78/2597, 3%). This suggests that the program is acceptable to most patients [18]. The themes and subthemes that we identified here also suggest that across all SDOH bands, patients share the same intentions to complete their FIT. The FQHC may have built trust through its multimodal FIT outreach program, as reflected in 2 subthemes: “Changed mind because of text message or fotonovela content” (n=46) and “Misplaced or ignored kit but interest is now rekindled” (n=26).

Being able to maintain engagement with patients was another positive program outcome. The positive sentiment and tone with which patients responded (“Plan to complete [their FIT] soon” and “Didn’t receive a FIT”) suggest that the frequency and level of messaging were acceptable and within patients’ tolerance. Other subthemes further confirmed and quantified prior positive patient feedback, including “Understands importance but life gets in the way” and “Putting off or avoiding because it is unpleasant.” The FQHC will work toward developing strategies to address these barriers [18]. Patients who remained hard to influence were represented in each of our SDOH bands, suggesting that continued attention to social needs is important in our screening efforts.

### Limitations

Although the texting program and fotonovela-incorporated barriers have been reported in the literature, there was no direct patient feedback on the materials.

One of the subthemes that received the most patient responses was “Returned kit already” (n=209). Patients may have returned their FIT before the program’s week 1 reminder text, with the FIT still in the mail or yet to be updated in the FQHC’s database.

When enrolling patients in the intervention group, we validated patient phone numbers as mobile phone numbers, but we did not validate the patient’s home address. Thus, it is possible that the information presented in the SDOH maps is inaccurate.

### Conclusions

For other FQHCs seeking to increase patient engagement with patients with the goal of completing fecal CRC screening, our findings suggest that culturally tailored text messaging can encourage patient engagement and ultimately FIT completion. Engagement can also generate insights into the gaps in patient care and barriers to behavior change. Intervention patients who replied provided actionable information for addressing gaps in care, such as mailing out kits never received. We also remain aware of barriers where patients understand the importance of their screening, but “life gets in the way.” We tried to address this through the fotonovela storyline; however, this barrier still persists and requires more work to understand how to bridge it.

The results of the thematic analysis of patient responses were positive overall, and there were no explicit objections to the fotonovelas. However, because this feedback was not linked at the individual patient level to whether a patient had clicked on the fotonovela, determining this rate and its relationship with screening rates is an area for future work. In addition, fotonovelas are a static asset that may require changes as time progresses. For example, the *Turning 50* fotonovela was updated to reflect the US Preventive Services Task Force recommendation to begin screening at the age of 45 years [24]. Collecting patient input from this newly eligible age group will be critical to identifying potentially different barriers to screening compared with those for patients aged ≥50 years [25].

Fotonovelas also have the potential to be developed to address other behaviors in the CRC screening pathway, such as responding to an abnormal result or preparing for a colonoscopy. It is also important to consider a unidirectional versus a bidirectional texting program, as the latter, with much more costly AI and natural language decision trees, may be unsustainable for community clinics. Areas for future work include the cost analysis of these texting program options.

Finally, to better support all patients undergoing CRC screening, we must continue to explore and test additional strategies to engage patients who did not respond to the program.

## Appendix

Multimedia Appendix 1 Addressing patient colorectal cancer screening barriers using tailored text messaging and fotonovelas informed by behavioral frameworks.

Multimedia Appendix 2 Theme 1: messages as a reminder.

Multimedia Appendix 3 Theme 3: expressing barriers.

## Abbreviations

AI: artificial intelligence
CRC: colorectal cancer
FIT: fecal immunochemical test
FQHC: Federally Qualified Health Center
NLU: natural language understanding
SDOH: social determinants of health
SES: socioeconomic status

## Data Availability

The data sets generated or analyzed during this project are available from the corresponding author upon reasonable request.
