# Supplementary material for: Tailoring a Text Messaging and Fotonovela Program to Increase Patient Engagement in Colorectal Cancer Screening in a Large Urban Community Clinic Population: Quality Improvement Project
Source: JMIR Cancer. 2023 Aug 10;9:e43024. doi: 10.2196/43024 (PMC10450532; doi:10.2196/43024)
Supplement: Multimedia Appendix 1 [file cancer_v9i1e43024_app1.docx]

**Table 1.** Addressing patient colorectal cancer screening barriers using tailored text messaging and fotonovelas informed by behavioral frameworks.

| Barrier or behavior that patient could exhibit | How behavioral change strategies were embedded into the program | How fotonovela and NLU^a^ addressed the behavior |
| --- | --- | --- |
| Lack of CRC^b^ awareness | 1. Tailoring to stage of change (inconsistent vs never completed) 2. Building health literacy about CRC | 1. Example patient text: “I don’t feel any pain, I don’t have any symptoms, why should I complete the test?”  - Example response from NLU: “The FIT^c^ test can help you detect colon cancer. It’s easy and highly valuable.” |
| Fear of colonoscopy (of pain or unknown) | 1. Information about procedure and health consequences | 1. Example response from NLU: “Returning the test will result in quick information about the outcome.”  - SMS text message system sends texts that walk through the steps and reasons to do it. |
| Fear of finding out about serious disease | 1. Decision balance—perceived susceptibility, severity, and benefits of screening 2. Anticipated regret 3. Comparative imagining of future outcomes | 1. Example patient text: “I also kind of fear ‘what if they find something?’ I feel like you can’t survive cancer... so I’d rather die without knowing I have it.”  - Response from NLU: “There are way more benefits than the trouble of doing it, it will give you peace of mind.... and there are things you can do if you find out early that you have cancer.” |
| Embarrassment of screening | 1. Health beliefs and machismo | 1. System sends texts that address that FIT envelopes are spill-proof and provide statistics about benefits of early screenings. |
| Lack of time for the use of health services | 1. Restructuring physical environment | 1. Text from NLU: “It’s quick and easy to do the FIT test. Here’s a suggestion—put it in plain sight so that it will be a reminder. In a place you cannot ignore.” |
| Procrastination | 1. Goal setting and action planning | 1. Phrase from fotonovela: “I’ll put it on my to-do list.” |
| Disinterest toward health | 1. Identify self as role model for others | 1. Phrase from fotonovela: “It’s a great way to take charge of my health as I enter into my 50s!” |
| Nonacceptance of biopsy | 1. Debunk myths and misperceptions 2. Gain-framed messaging | 1. Phrase from fotonovela: “My doctor said if it is found early, cancer can be treated and cured.” |
| Lack of self-care | 1. Promote positive self-talk | 1. Phrase from fotonovela: “I want to protect my health. To live many more years. To be around for my family.” |
| Low self-esteem | 1. Boost motivation and self-efficacy 2. Verbal persuasion | 1. System sends a link to a picture which shows the steps needed. 2. Text from NLU: “Do it the right way—so the test results are more accurate.” |

^a^NLU: natural language understanding.

^b^CRC: colorectal cancer.

^c^FIT: fecal immunochemical test.
