# Supplementary material for: Tailoring a Text Messaging and Fotonovela Program to Increase Patient Engagement in Colorectal Cancer Screening in a Large Urban Community Clinic Population: Quality Improvement Project
Source: JMIR Cancer. 2023 Aug 10;9:e43024. doi: 10.2196/43024 (PMC10450532; doi:10.2196/43024)
Supplement: Multimedia Appendix 2 [file cancer_v9i1e43024_app2.docx]

**Table 2.** Theme 1: messages as a reminder.

| Subtheme | Example Quote |
| --- | --- |
| **Returned kit already (n=209)** | |
| These patients returned their kits after receiving the reminder or, in some cases, even before the reminder messages. | - “I totally forget it when I go to use the toilet [1 week later] I sent mine in already.” [Male, English speaker, age 57 years, very high SDOH^a^ impact] - “No need to check back, I sent it in already.” [Male, English speaker, age 62 years, very high SDOH impact] - “Yes, I already took it to the clinic.” [Female, Spanish speaker, age 62 years, very high SDOH impact] - “I turned it in on October 13 ALREADY.” [Female, English speaker, age 59 years, Unknown SDOH impact] |
| **Plan to complete soon (n=88)** | |
| These patients viewed the reminder as a call to action. They were not averse to completing the screening but needed a nudge to move forward.  Sentiment was positive, and a few patients apologized for the delay in getting this done. | - “Yes, we totally forgot. Will do. Thanks!” [Female, English speaker, age 73 years, low SDOH impact] - “No I haven't yet I have one though, I'll get it done asap.” [Male, English speaker, age 57, very high SDOH impact] - “Yes, thanks, I’ll return it, don’t worry.” [Female, Spanish speaker, age 68 years, very high SDOH impact] - “I will take care of it I have one. sorry for the inconvenience thank you.” [Male, English speaker, age 59 years, unknown SDOH impact] - “Ok I will send it as soon as possible Thank you.” [Male, Spanish speaker, age 62 years, very high SDOH impact] - “Yes but I can send the same [sample] tomorrow to the laboratory*.”* [Female, Spanish speaker, age 60 years, high SDOH impact] |
| **Will pick up at clinic (n=25)** | |
| Others (who did not already have a kit) were keen to move things along by picking up an FIT kit at a nearby clinic instead of waiting to receive one in the mail. | - “No...I can drive there & pick one up. [Male, English speaker, age 62 years, medium SDOH impact] - “I’m going in to [FQHC] today, I’ll pick one up.” [Female, English speaker, age 64 years, high SDOH impact] - “I have not received one in the mail. I actually have an appointment this afternoon. Can I pick one up?” [Female, English speaker, age 51 years, Unknown SDOH impact] |
| **Will drop off at doctor’s office (n=8)** | |
| These patients might be more comfortable with delivering the kit to their doctor.  The provider might have encouraged or recommended the screening and would be able to answer any questions relating to results of the screening). | - “I have an appt with Dr. [Redacted] on the 10/25, and will drop off the test at that time.” [Female, English speaker, age 68 years, medium SDOH impact] - “I was with my doctor and there they gave me the paper for the test so that I could take it to my next appointment which is this month.” [Female, Spanish speaker, age 70 years, very high SDOH impact] - “I was told to bring it in person.” [Male, English speaker, age 61 years, unknown SDOH impact] - “You mean the brush stool kit? I will bring with me on my doctor’s visit tomorrow.” [Male, English speaker, age 55 years, high SDOH impact] |
