# Supplementary material for: Tailoring a Text Messaging and Fotonovela Program to Increase Patient Engagement in Colorectal Cancer Screening in a Large Urban Community Clinic Population: Quality Improvement Project
Source: JMIR Cancer. 2023 Aug 10;9:e43024. doi: 10.2196/43024 (PMC10450532; doi:10.2196/43024)
Supplement: Multimedia Appendix 3 [file cancer_v9i1e43024_app3.docx]

**Table 4**. Theme 3: expressing barriers.

| Subtheme | Example Quote |
| --- | --- |
| **Did not receive an FIT kit (n=290)** | |
| These patients did not receive the FIT kit in the mail and were requesting another kit to complete and send back.  The tone was generally polite and positive. | - “I don’t have a package 📦 for the test.” [Male, Spanish speaker, age 60 years, very high SDOH^a^ impact] - “No please send another. Thank you.” [Male, Spanish speaker, age 62 years, high SDOH impact] - “I have not received the FIT kit. Please mail it to me and I’ll complete it.” [Female, English speaker, age 63 years, unknown SDOH impact] - “Yes but I need the kit please 🙏” [Female, Spanish speaker, age 63 years, very high SDOH impact] - “Please mail me one out thank you 😀” [Female, English speaker, age 59 years, medium SDOH impact] |
| **Have health or mobility issues (n=16)** | |
| These patients had health issues and needed assistance, more time, or a good reason to complete the test. | - “Well it might be quick and easy for you that have 2 working arms and legs. It’s difficult for me to balance.” [Female, English speaker, age 56 years, very low SDOH impact] - “I had a car accident and I had surgery on my leg and it is very painful*.*” [Female, Spanish speaker, age 51 years, very high SDOH impact] - “No, I had my fibula broken so I have been in a recovery home.” [Male, English speaker, age 51 years, medium SDOH impact] - “I already have cancer.” [Female, Spanish speaking, age 57 years, very high SDOH impact] |
| **Planning to get a different colon screening (n=13)** | |
| The FIT test was not appropriate in these cases because they had recently completed a colonoscopy (or had one scheduled soon). | - “I already had a colonoscopy last year.” [Female, Spanish speaker, age 61 years, very high SDOH impact] - “Had a colonoscopy last month. They said I didn’t need to do that until next year!” [Female, English speaker, age 59 years, very high SDOH impact] - “I had a colon exam and bioscopy in November 2017.” [Male, Spanish speaker, age 64, High SDOH impact] - “I have my first appointment at the end of November for an endoscopy and colonoscopy.”[Female, English speaker, age 58 years, medium SDOH impact] |
| **Not interested in the screening (n=10)** | |
| These patients were not open to influence or persuasion and made it clear that they would not do the test. | - “I don’t want you to send me one.” [Female, Spanish speaking, age 57 years, very high SDOH impact] - “I don’t want to do it.” [Female, Spanish speaking, age 58 years, very high SDOH impact] |
| **Putting off or avoiding because it is unpleasant** (**n=9)** | |
| These patients found the test disgusting or unpleasant but might also be confusing the FIT test with preparation for a colonoscopy. | - “I can’t stomach drinking the solution that clears the intestines. It is a painful process that my body won’t allow me to go through with it.” [Male, English speaker, age 60 years, very high SDOH impact] - “Because it disgusts me to see that test, I’m going to do it.” [Female, Spanish speaker, age 53 years, medium SDOH impact] |
| **Understands importance but life gets in the way (n=6)** | |
| These patients took the time to explain why they were putting off completing the test and shared a mix of family concerns and other commitments. | - “I am so busy packing I am moving to a smaller apt. Everything is everywhere. I will worry about this after the new year” [Female, English speaker, age 62 years, low SDOH impact] - “Just haven't had the time yet... Been busy with family concerns.” [Female, English speaker, age 61 years, high SDOH impact] - “No, I have been taking care of my mom I’m sorry” [Male, English speaker, age 61 years, very low SDOH impact] - “I’m focused on a professional exam. Excuse me, tonight I complete it.” [Male, Spanish speaker, age 67 years, high SDOH impact] - “I haven’t had a chance to see it. I’ve got other big worries right now financially and I’m on a mission, I’ll get back with you shortly.” [Male, English speaker, age 52 years, high SDOH impact] |
